# Moving from reactive response to proactive prevention of emerging infectious diseases: Socio-ecological systems mapping in the Democratic Republic of the Congo

**DOI:** 10.1371/journal.pgph.0005400

**Published:** 2025-12-16

**Authors:** Marc K. Yambayamba, Marlène Metena, Rolly Paku, Chris Lutonda, Florence Ngolole, Emile F. Bongono, Sheila Makiala-Mandanda, Justin Masumu, Simon R. Rüegg

**Affiliations:** 1 Section of Epidemiology, Vetsuisse Faculty, University of Zurich, Zurich, Switzerland; 2 Department of Epidemiology and Biostatistics, Kinshasa School of Public Health, University of Kinshasa, Kinshasa, Democratic Republic of the Congo; 3 Faculté de Médecine Vétérinaire, Université Pédagogique Nationale, Kinshasa, Democratic Republic of the Congo; 4 Projet DOPERAUS, Institut National des Recherches Biomédicales, Kinshasa, Democratic Republic of the Congo; 5 Université Gamal Abdel Nasser de Conakry, Conakry, Guinée; 6 Département de Biologie Médicale, Faculté de Médecine, Université de Kinshasa, Kinshasa, Democratic Republic of the Congo; 7 Network for Ecohealth and One Health (NEOH), Brussels, Belgium; University of St Andrews, UNITED KINGDOM OF GREAT BRITAIN AND NORTHERN IRELAND

## Abstract

Emerging infectious diseases such as Ebola and Mpox pose significant public health challenges in the Democratic Republic of the Congo (DRC). Effective prevention policies require a clear understanding of the socio-ecological systems (SES) in which these diseases emerge. This study examined the SES influencing emerging infectious disease prevention in the DRC through five participatory modelling workshops conducted at national, provincial, and community levels using causal loop diagrams (CLDs). Participants were selected through stakeholder analysis to ensure cross-sectoral representation. A structured process guided the co-creation of integrated system maps, beginning with disease-specific models and culminating in validated shared maps. A total of 162 stakeholders participated across the workshops, most of whom were affiliated with government institutions (83%), with smaller proportions from civil society, academia, and technical assistance organizations. The Agriculture and Animal Health sector represented 36% of participants, followed by Human Health (31%) and Environmental Health (13%). Most participants had over 10 years of experience. Analysis of the CLDs revealed that while the number of infected individuals remained the central driver triggering feedback responses, the mechanisms of influence differed by governance level. National and provincial systems were shaped by public investment in One Health systems, political commitment, and governance capacity, whereas community-level dynamics were dominated by socio-economic conditions, hunting practices, and local sensitization. Overall, the findings highlight that current governance remains largely reactive, emphasizing response over prevention. Strengthening One Health governance will require a shift toward proactive health promotion supported by institutionalized coordination, sustained investment, and inclusive community engagement.

## Background

The Democratic Republic of the Congo (DRC) faces recurrent emerging infectious diseases (EIDs) [1]. These include Ebola outbreaks, Mpox, COVID-19, yellow fever and many others. Since the first outbreak in 1976, the country has faced 16 Ebola outbreaks focused on Equateur, Kasai and Kivu regions [2–4]. From the first case of Mpox in 1970, the disease was endemic in Sankuru and Tshopo regions before extending to 22 provinces of the 2024 outbreak [2,5]. The latter was declared a Public Health Emergency of International Concern by the World Health Organization (WHO) and Public Health Emergency of Continental Security by Africa Centres for Disease Control and Prevention (Africa CDC) [6,7].

In recognition of the complex and interrelated drivers of disease outbreaks, the DRC already established a National One Health (OH) Platform in 2011, aimed at fostering multisectoral collaboration between the health, agriculture, environmental, and wildlife sectors [8]. This platform was designed to improve the prevention, early detection, and response to zoonotic and other EIDs, reflecting a growing recognition that human, animal, and ecosystem health are closely linked [8]. The OH approach seeks to address this interconnectedness by promoting cross-sector coordination, communication, information sharing, and joint action, thereby enhancing the country’s capacity to address EIDs more effectively [9]. However, the Joint External Evaluation (JEE) of the International Health Regulations (IHR) identified persistent gaps. These included the need for stronger coordination mechanisms, more robust surveillance systems, and improved response capacities at all levels of governance [10].

Effective prevention of EIDs requires a clear understanding of the socio-ecological systems (SES) in which they arise [11]. Outbreaks such as Ebola and Mpox are shaped by a complex interplay of environmental, social, and economic factors. To address these challenges, it is important to use appropriate frameworks to analyse these interconnected components [12]. Causal loop diagrams (CLD), initially developed in business and ecology, have become a useful tool in public health to examine complex systems [13] particularly in tackling infectious diseases [14–18]. Complex systems often exhibit non-linear behaviours driven by feedback loops mechanisms through which changes in one part of the system influence and are influenced by other components over time [12]. CLDs offer a visual method for mapping these feedback loops, causal relationships, and dynamic interactions within systems, making them well-suited for understanding disease dynamics [13].

A key strength of CLDs is that they can be used in participatory approaches, which enables stakeholders to collaboratively build a shared mental model of the challenge they aim to address [13,16]. At the same time, they allow representing material, immaterial, measurable and non-measurable variables in the system and their relationships. A mental model reflects an individual’s internal representation of the external world, and when CLDs are co-produced in group settings, they allow integrating diverse perspectives [14,19]. By mapping causes, effects, and interactions, stakeholders can identify leverage points, barriers, and possible unintended consequences of public health policies [12,20]. In public health, this method has been used to promote multisectoral collaboration by engaging stakeholders from sectors like public health, environmental and animal health, and community organizations [13,18]. Applied in an inclusive process, the method improves the development of more holistic interventions and encourages greater problem and solution ownership among participants. The use of CLDs helps guide system-level interventions that are necessary for addressing the complex web of factors behind infectious disease outbreaks. In this study, we used co-produced causal loop diagrams (CLDs) to understand the SES for EID prevention in the DRC across governance levels, focusing on identifying leverage points for action and feedback loops.

## Methods

### Ethical statement

The study was approved by the Institutional Review Board of the University of Kinshasa School of Public Health (ESP/CE/161B/2022). An informed verbal consent was obtained from all participants, ensuring that they were aware of the study’s objectives, methods, and potential risks and benefits.

### Inclusivity in global research

Additional information regarding the ethical, cultural, and scientific considerations specific to inclusivity in global research is included in the Supporting Information (S1 Checklist).

### Study design, Setting and study period

We conducted a participatory mixed-methods study to better understand the SES in which infectious diseases occur, are controlled and prevented in the DRC.

The DRC is the second largest African country, bordered by nine countries, with a population exceeding 100 million [21,22]. The country is divided into 26 provinces, created during a decentralization reform in 2015 [22,23]. Each province has a degree of administrative autonomy, with a government responsible for managing services like health, education, infrastructure, and security [22]. This decentralization aims to improve governance, resource allocation, and development by bringing decision-making closer to local populations. Kinshasa is the capital and a standalone province, reflecting its unique administrative, economic, and political importance [24,25]. Provinces outside Kinshasa are further divided into a total of 145 territories [22], which represent the rural and semi-urban administrative units often encompassing smaller towns, villages, and rural communities. Each territory has its administrative leadership responsible for implementing provincial policies, collecting data, and managing services in coordination with provincial authorities [25]. In urban areas, particularly within major cities, territories are replaced by communes. Communes represent smaller administrative divisions within cities, focusing on urban-specific issues like public transportation, housing, and sanitation. Large cities may have multiple communes managed by local officials.

We held a series of two-day workshops at the national level in Kinshasa and the provincial level in Kinshasa and Equateur provinces, and at the communal level in Kinshasa between November 2022 and August 2023 (see S1 Text for the detailed schedule).

### Participants

We used stakeholder analysis to ensure broad and balanced participation across Human Health, Agriculture and Animal Health, Environmental Health sectors, and community stakeholders. Participants were eligible if they were involved in emerging infectious disease prevention, surveillance, or response activities within their institutions. They worked for government institutions, civil society organizations, and technical support (donor) organizations at national, provincial, and communal levels. At national level, two workshops were held one with the national One Health platform (“Commission de Coordination Une Santé”, CCUS) and one with further national stakeholders in One Health (designated “National” in this article). Further workshops were held at the provincial level of Kinshasa and Equateur (Mbandaka) and one at the communal level of Kinshasa with participants from four randomly selected communes (Bandalungwa, Kalamu, Kimbanseke, and Masina).

### Mapping workshops

The participatory workshops were conducted using a Group Model Building (GMB) approach [26]. This system thinking methodology enables stakeholders to collaboratively conceptualize and map the dynamics underlying complex health challenges [27]. The goal was to elicit participants’ collective understanding of zoonotic disease prevention and identify feedback loops and leverage points using the One Health approach. The facilitation process followed established GMB scripts adapted from the Scriptapedia repository [28]. These scripts provided structured guidance for each activity, ensuring consistency, transparency, and reproducibility across sessions. Each two-day workshop followed a stepwise process, beginning with an introduction to One Health and systems thinking concepts to establish the theoretical framework necessary for the modelling process. Participants were introduced to system thinking especially causal loop diagramming and behaviour over time using practical examples before its application to infectious disease control. In every workshop Ebola, Mpox, COVID-19, rabies were proposed as a modelling subject. A group of 4–5 persons was assigned to each disease. To facilitate high diversity of perspectives, each group was composed of participants from different sectors. Each workshop was supported by a facilitation team of five trained facilitators. One facilitator served as the lead moderator, introducing each session, providing instructions, and guiding plenary discussions. The remaining four facilitators were each assigned to a small group to provide hands-on support throughout the modelling activities. Their roles included ensuring balanced participation, clarifying methodological questions, and maintaining focus on the exercise objectives. A detailed description of the workshop methodology is provided in Supplemental materials (see S1 Text for the detailed workshop methodology).

### Data analysis

Participant characteristics were described using proportions and percentages and used the mean and standard deviation for age and professional experience after assessing for normality. The digital map was exported from the Cmaps software as a list of propositions in a text file, imported into R identification of feedback loops [29]. To better understand the dynamics of the system, we identified and visualized reinforcing and balancing feedback loops using Vensim [30]. The first work toward exponential growth or potentially exponential decline and the second to equilibrium in the system [31]. We provide a description of each causal loop diagram identified.

## Results

### Participants

Overall, 5 workshops were conducted with a total of 162 participants. Most participants were affiliated with government institutions (83%), with smaller proportions from civil society, academia, and technical assistance organizations. The overall gender distribution was predominantly male (76%), and the average professional experience was 15 years, indicating the inclusion of senior professionals across sectors and governance levels. Overall, the Agriculture and Animal Health sector represented 36% of participants, followed by the Human Health sector with 31%, and the Environment Health 13% respectively (Table 1).

**Table 1:** Participants characteristics.

Characteristics	Overall (%) N = 162	CCUS (%) N = 31	National (%) N = 33	Kinshasa province (%) N = 37	Equateur province (%) N = 31	Commune Kinshasa (%) N = 30
**Age (Mean, SD)**	44 (9)	44 (10)	42 (11)	43 (7)	46 (10)	44 (7)
**Gender**						
Female	39 (24.0)	8 (26.0)	10 (30.0)	9 (24.0)	1 (3.0)	11 (37.0)
Male	123 (76.0)	23 (74.0)	23 (70.0)	28 (76.0)	30 (97.0)	19 (63.0)
**Participants sector**						
Agriculture and Animal Health	58 (36.0)	5 (16.0)	11 (33.3)	22 (59.5)	10 (32.0)	10 (33.3)
Environmental Health	21 (13.0)	7 (22.5)	3 (9.1)	3 (8.0)	4 (13.0)	4 (13.4)
Human Health	51 (31.0)	7 (22.5)	16 (48.5)	5 (13.5)	13 (42.0)	10 (33.3)
Others*	32 (20.0)	12 (39.0)	3 (9.1)	7 (19.0)	4 (13.0)	6 (20.0)
**Institution type**						
Government	135 (83.3)	21 (68.0)	31 (94.0)	31 (84.0)	28 (90.0)	24 (80.0)
Civil society/NGOs	12 (7.4)	0 (0)	1 (3.0)	3 (8.0)	2 (7.0)	6 (20.0)
Higher education/Universities	9 (5.6)	8 (26.0)	0 (0)	0 (0)	1 (3.0)	0 (0)
Technical assistance/donors	6 (3.7)	2 (6.0)	1 (3.0)	3 (8.0)	0 (0)	0 (0)
**Professional experience (Mean, SD)**	15 (8)	14 (10)	14 (9)	15 (7)	16 (8)	14 (7)

*Note: “Others” include participants from the Ministry of Interior, Ministry of Media and Communication, and related administrative offices.

### Map of “National” workshop

**Fig 1:**
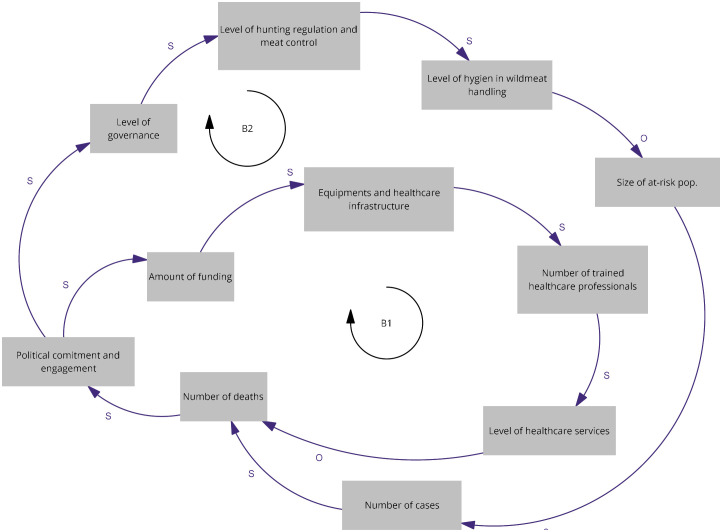
Relevant causal loops of the diagram co-produced at the “National” workshop. The arrows denote causal links with s in the same direction and o in opposite direction. R denote reinforcing loops and B balancing loops.

We identified two balancing loops, B1 and B2, as shown in Fig 1. Both originate from political commitment and engagement, which is primarily triggered by the number of deaths. In balancing loop B1, an increase in deaths stimulates greater political engagement, leading to increased funding for healthcare infrastructure, equipment, and workforce training. These investments improve healthcare services and contribute to a subsequent reduction in deaths. However, as mortality declines, political attention and financial allocations also diminish, creating a balancing mechanism that stabilizes investment around a socially tolerated level of mortality. In balancing loop B2, political commitment enhances governance capacity, influencing hunting regulations and the control of wild animal meat. Improved enforcement and hygiene during meat handling reduce the size of the population at risk, lowering case numbers and deaths. As the situation improves, funding and commitment again decline, balancing investment with disease occurrence and mortality.

### Map of the CCUS workshop

**Fig 2:**
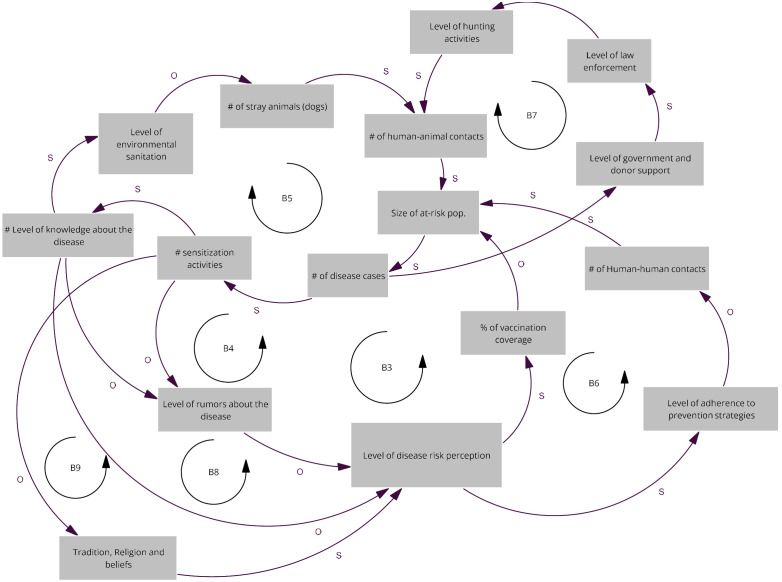
Relevant causal loops of the diagram co-produced at the CCUS workshop. The arrows denote causal links, with s in the same direction and o in opposite direction, R denote reinforcing loops and B balancing loops.

We identified two key driving factors: an increase in the number of deaths, and the level of response from both the government and donors. A rise in deaths triggers intensified awareness-raising activities and greater support from government and donors. Balancing loops (B3 to B9) which all impact on the size of the at-risk population either via risk perception and consequent vaccination coverage (B3, B4, B8 and B9), by reducing human-to-animal contacts (B5 and B7), and human-to-human contacts (B6). These actions lead to improved prevention and control measures, which subsequently reduce the number of deaths. As mortality declines, however, attention and resources from actors tend to decrease creating balancing feedback loops (Fig 2).

### Map of the Kinshasa province workshop

**Fig 3:**
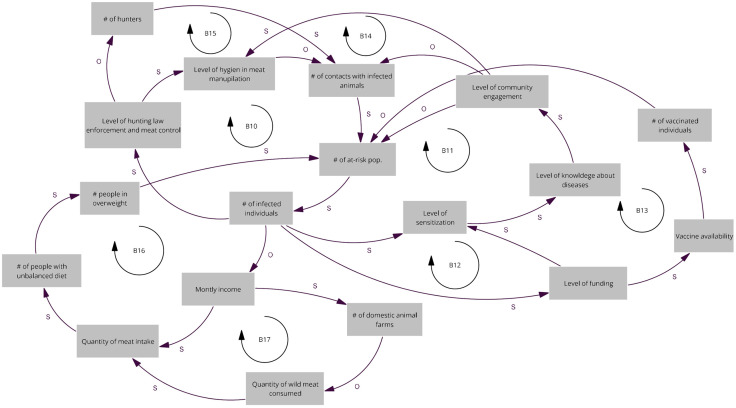
Relevant causal loops of the diagram co-produced at the Kinshasa province workshop. The arrows denote causal links, with s in the same direction and o in opposite direction. R denote reinforcing loops and B balancing loops.

This map is primarily driven by the number of infected individuals, which activates multiple balancing mechanisms within the system. An increase in infections can influence monthly income, which in turn affects wild animal meat consumption. Higher income levels reduce dependence on wild meat, thereby lowering exposure to zoonotic risks. Similarly, an increase in cases can stimulate sensitization campaigns and funding support, both of which contribute to reducing the size of the at-risk population. Participants also highlighted the role of domestic animal farming: as the number of farms increases, the local supply of domestic meat rises, reducing the demand for wild animal meat. However, improved income and greater meat availability may lead to dietary imbalances and obesity, potentially increasing vulnerability to disease (B16 and B17). In parallel, a higher number of infections can prompt stronger hunting law enforcement, decreasing the number of hunters and consequently the human–animal contacts (B10 and B15). These measures also enhance hygiene practices in wild meat handling (B10). Furthermore, increased infection levels can trigger additional funding, improving access to medical countermeasures such as vaccination (B12 and B13). The rising number of cases can also heighten community sensitization (B11), strengthening knowledge about disease transmission and reducing the at-risk population, or enhance community engagement (B14), leading to better hygiene in meat handling and processing (Fig 3).

### Map of the Equateur province workshop

**Fig 4:**
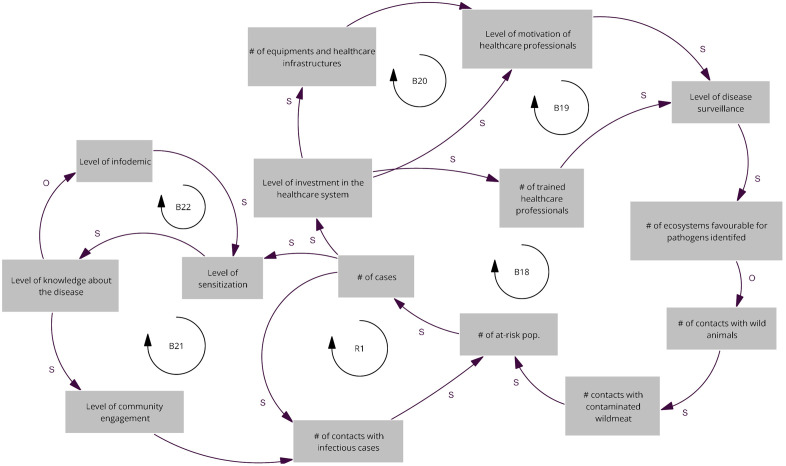
Relevant causal loops of the diagram co-produced at the Equateur province workshop. The arrows denote causal links, with s in the same direction and o in opposite direction. R denote reinforcing loops and B balancing loops.

The reinforcing loop (R1) reflects how an increase in the number of infected individuals, combined with limited diagnostic capacity and restricted access to health care, leads to more frequent contacts with infectious cases. This, in turn, expands the population at risk and reinforces the number of new infections. The five balancing loops (B18, B19, B20, B21 and B22) represent feedback acting through improvements in the health system and surveillance and level of sensitization. As the number of cases increases, it stimulates greater public investment in health services with the acquisition of medical equipment (B20), including the training and motivation of the health workforce (B19). These investments enhance surveillance activities, improve the identification of ecosystems favourable to pathogen emergence, and promote actions that limit hunting and reduce human–wildlife contact (B18). The same increase in number of cases triggers sensitization activities to improve community knowledge about the disease and their engagement (B21) and impacts the level of infodemic (B22) as seen in Fig 4.

### Map of the Kinshasa commune workshop

**Fig 5:**
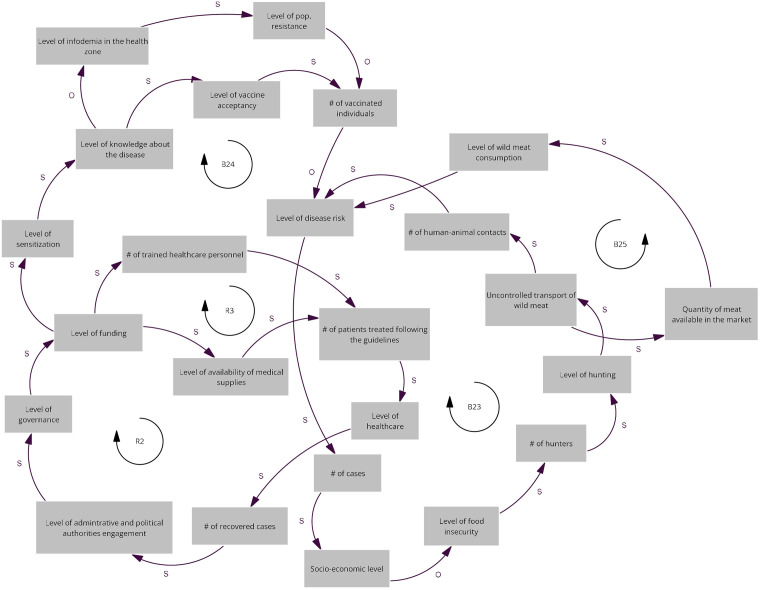
Relevant causal loops of the diagram co-produced at the Kinshasa commune workshop. The arrows denote causal links, with s in the same direction and o in opposite direction. R denote reinforcing loops and B balancing loops.

We identified two reinforcing loops (R2 and R3) and three balancing loops (B23, B24, and B25) (Fig 5).

The reinforcing loops (R2 and R3) operate through improvements in healthcare performance. As the number of patients treated according to clinical guidelines increases, the overall level of healthcare and the number of recovered cases also rise. These outcomes strengthen political and administrative engagement, enhance governance, and stimulate public funding, which in turn support the training of health personnel and the availability of medical supplies further reinforcing the health system’s capacity to respond. The balancing loops (B23, B24, and B25) act through socio-economic and behavioural feedback that counter disease spread. An increased level of disease risk and a higher number of cases negatively affect the socio-economic status of residents, heighten food insecurity, and influence hunting activities and the uncontrolled transport of wild meat. Two of these loops (B23 and B24) operate through direct human–animal contact and wild meat consumption, respectively, while the third (B25) acts via community sensitization, which improves knowledge and adherence to preventive practices, thereby reducing exposure and stabilizing the system (Fig 5).

## Discussion

This study identified a set of interrelated drivers that shape the dynamics of zoonotic disease prevention and response across governance levels. The analysis revealed that while the number of infected individuals remains the central driver triggering feedback responses, the mechanisms through which this influence operates differ by level. At the national and provincial levels, system behaviour was primarily shaped by public investment in health systems (human, animal and environment), political and administrative engagement, and governance capacity, which reinforce or constrain the institutional response to outbreaks (B1, B2, B7, B10, B13, B15, B18, B19 and B20). In contrast, at the community level, socio-economic conditions, hunting practices, wild meat consumption, and community sensitization emerged as dominant behavioural and ecological drivers that determine population exposure and resilience (B16, B17, B23 and B25). Despite these differences, a common pattern was the balancing role of community engagement and awareness and the reinforcing effect of governance and funding cycles across all levels (B3, B4, B5, B6, B8, B9, B11, B12, B14, B21, B22, and B24). We identified three reinforcing loops, R1 reflects how an increase in the number of infected individuals, combined with limited diagnostic capacity and restricted access to health care, leads to more frequent contacts with infectious cases. R2 and R3 act through improvements in healthcare performance and investment in the healthcare system. Together, these findings underscore the current system focused on reactive response to outbreaks than proactive investment in disease prevention.

The difference between the national, provincial levels aligns with global evidence that centralized bodies often emphasize upstream determinants and broad policy levers [32]. Meanwhile, communal-level priorities were more localized with contextual factors that affect individual health behaviours and risks. These disparities reveal the limitations of top-down approaches in responding to local realities. In line with previous studies, our results suggest that decentralization must be coupled with strengthened local capacity and involvement of communities in the decision space [33–36]. For instance, communal-level concerns about socioeconomic level and behaviours point to areas where national health strategies must better engage with social and political determinants of health. In the DRC, 85% of the population lives below the poverty line (USD 3 per day), according to the World Bank [37], limiting their access to healthcare services and leading to food insecurity and reliance on risky behaviours such as wild animal meat hunting [38–40].

Political commitment was the key driver shaping public investment in health systems (human, animal and environment), political and administrative engagement, and governance capacity, which reinforce the institutional response to outbreaks. Consequently, political commitment appeared reactive instead of preventive, with increased attention following crisis events and declining interest once the immediate threat subsided. Political commitment, for the purposes of this analysis, is defined as the demonstrated intent and sustained action of political institutions or leaders to address specific public health challenges through the development and implementation of policies, mobilization of resources, and active public communication. The findings underscore a broader challenge in the DRC’s public health landscape: public health including disease prevention efforts remain heavily dependent on responsive external funding [41]. As observed in other global health domains, including primary healthcare, achieving effective prevention requires a shift from crisis-driven response to sustained investment in health, political leadership, and long-term institutional commitment [42–44]. Without such changes, health systems remain vulnerable to recurring outbreaks with sporadic high burdens that strain limited resources, or donor disengagement within geopolitical dynamics, which erode public trust [1,45]. Notably, two reinforcing loops (R2 and R3) in the Kinshasa communal-level model differed in framing. Rather than linking political commitment to epidemiological indicators such as case numbers or populations at risk, they connected it to improvements in the quality of care and service delivery. This framing suggests a perception that visible, positive health outcomes such as increased recovery rates or improved community satisfaction are stronger incentives for political engagement and funding than crisis metrics. While this framing may not fully reflect current realities of centralized and reactive governance in the DRC, it reveals local actors’ normative expectation that sustained improvements in service delivery should drive political attention and funding.

A common pattern was the balancing role of community engagement and awareness. Risk perception, shaped by knowledge, exposure to misinformation, and cultural beliefs, influence uptake of preventive behaviours, such as vaccination, safe burial practices, hygiene, or early care-seeking. Previous outbreaks of Ebola virus disease have shown how cultural practices such as washing or touching the deceased during funerals can amplify transmission [46]. Similarly, traditional understandings of illness, including beliefs in supernatural causation or reliance on herbal remedies, may delay or replace biomedical care-seeking, particularly in rural or underserved settings [47,48]. In the DRC, sensitization activities remain largely reactive and predominantly donor funded as part of outbreak response plans [41,49]. Strengthening local ownership, mobilizing trusted community actors, and sustaining risk communication beyond emergencies could improve engagement and adherence to preventive measures. The reliance on traditional knowledge emphasises the importance of integrating various knowledge systems into a One Health approach and suggests that there may be similar potential synergies between biomedical and traditional health practices as demonstrated in Peru during the COVID epidemic [50].

The diversity of map components spanning the human, animal, environmental, and socioeconomic sectors underscores the importance of adopting a One Health approach in disease prevention. It also revealed that stakeholders recognize the interdependence between human health and broader ecological factors, as evidenced by the inclusion of variables such as the number of dog bites, human-animal contacts, consumption of wild animal meat, and the presence of ecosystems favourable to disease transmission. These findings suggest a strong awareness among workshop participants of the multifactorial drivers of disease and the pivotal role that human-animal-environment interfaces play in shaping disease dynamics. Such complexity cannot be addressed effectively by siloed interventions [51]. Instead, multisectoral and multidisciplinary collaboration including active community engagement is essential for developing a shared understanding of disease systems and designing contextually relevant interventions [9]. Strengthening One Health coordination structures and institutionalizing collaborative platforms will be pivotal to operationalizing this integrated vision. In line with prior evidence [52] this study reinforces the call for institutional investment in One Health mechanisms as a foundation for more resilient and responsive health systems [53,54].

A previous study has used CLDs to understand factors driving zoonotic spillover risk in countries including the DRC using stakeholder interviews and includes some of the factors considered here like human-human and human-animal contact [55]. To our knowledge, this is the first application of CLDs in a context of group model building to investigate the systemic factors underpinning repeated disease outbreaks in the DRC. More studies have typically used pathogen-specific or risk factor–based approaches that focus on isolated variables rather than system-wide interactions. The format of the workshops based on group modelling provided an opportunity for actors to enhance their collaboration and consensus while building a common understanding of the system in which they operate. Using CLD allowed participants to rethink the links between components of their systems and their dynamic interactions.

Nonetheless, several limitations should be noted. First, CLDs are based on the perceptions and experiences of participating stakeholders. This method is inherently subjective and may exclude relevant factors not recognized or emphasized by participants. Second, CLDs are qualitative and descriptive tools. They are valuable for identifying feedback mechanisms and hypothesizing about system behaviour. The method is limited for estimating the strength of relationships or predicting outcomes such as can be done with fuzzy cognitive mapping or system dynamics modelling [56,57]. In contrast, CLD’s allow for the consideration of non-measurable variables and can be operationalized in data scarce settings. Finally, the level of participant engagement and their role within the system may also have influenced the representation of certain factors in the maps [13,31]. However, the workshop facilitators made an effort to manage group dynamics by providing regular feedback to the group and encouraging different perspectives.

## Conclusion

The results highlight the critical role of integrating community-level concerns, particularly socio-economic status, alongside national and provincial political commitment and funding, in shaping prevention of emerging infectious diseases across all levels of governance. Sustaining engagement with traditional and community opinion leaders beyond outbreak responses can positively influence community risk perception and uptake of preventive measures. More broadly, One Health governance should shift from reactive response to recurrent outbreaks toward proactive investment in health promotion, supported by dedicated infrastructure. Regular modelling workshops can help update the evidence base in the context of a rapidly changing system and support both policy formulation and evaluation. Additionally, these workshops can serve as a trust-building exercise across sectors, fostering collaboration and shared understanding among stakeholders.

## Supporting information

S1 Checklist Inclusivity in global research questionnaire. (PDF)

S1 Text Workshop schedules and detailed methodology. (PDF)
