# Supplementary material for: Moving from reactive response to proactive prevention of emerging infectious diseases: Socio-ecological systems mapping in the Democratic Republic of the Congo
Source: PLOS Glob Public Health. 2025 Dec 16;5(12):e0005400. doi: 10.1371/journal.pgph.0005400 (PMC12707627; doi:10.1371/journal.pgph.0005400)
Supplement: S1 Text — (PDF) [file pgph.0005400.s002.pdf]

Supplemental B

Group model building methodology

The participatory workshops were conducted using a Group Model Building (GMB) approach[1]. This system thinking methodology enables stakeholders to collaboratively conceptualize and map the dynamics underlying complex health challenges [2]. The goal was to elicit participants' collective understanding of zoonotic disease prevention and identify feedback loops and leverage points using the One Health approach. Participants were acquainted with the model-building methodology and its application to infectious disease control. In every workshop Ebola, Mpox, COVID-19, rabies were proposed as a modelling subject. The facilitation process followed established GMB scripts adapted from the Scriptapedia repository [3]. These scripts provided structured guidance for each activity, ensuring consistency, transparency, and reproducibility across sessions. Preparatory meetings were held with research team members to outline the workshop objectives and establish the roles and responsibilities. The two-day workshops started with introducing One Health and systems thinking concepts including Behaviour Ober Time (BOT) and Causal Loop Diagramming using practical examples for participants to establish the theoretical framework necessary for the modelling process. Each workshop was facilitated by at least 5 facilitators, while one was presenting, the remaining four were attached to each group to manage the group dynamics, answer questions or provide clarification when needed by participants. Apart from a preparatory phase, the GMB workshop was divided into 4 phases described below.

Phase 1: Individual Mapping

During the individual mapping phase, participants identified tangible and intangible variables that influence the dynamics of disease transmission, surveillance, control and prevention of the

assigned disease within the social-ecological system. These variables were documented on paper notes for later use in the group modelling phase.

Phase 2: Small Group Mapping

A group of 4-5 persons was assigned to each disease. To facilitate high diversity of perspectives, each group was composed of participants from different sectors. Each group was tasked to integrate the variables identified in the individual models into a common model of the assigned disease system. This phase included discussion of variables within each group to achieve consensus and documenting the variables and attaching them to a flip chart for visualization. The next step was to identify causal relationships between the variables and representing them using causal loop diagrams (CLD)[4]. We used standards CLD notation, a Similar "S" arrow from A to B meaning that A and B change in the same direction and an opposite "O" arrow between A and B meaning they change in an opposite direction (23). Lastly, each group presented the final map to the plenary for other participants' feedback and further refinement. Disagreements were managed through facilitated discussion and consensus-building. Facilitators encouraged participants to explain differing perspectives and collaboratively agree on causal relationships. When differing views persisted, alternative linkages were noted for consideration during the plenary integration session, where the entire group discussed and agreed on the most representative formulation.

Phase 3: Plenary Mapping

All variables from the 4 or 5 small groups were integrated into a single map presented on a large paper chart. This was followed by an iterative review process in which each group spent 3-5 minutes reviewing the variables to identify and remove duplicates and structure the map by clustering related concepts. This process continued until no further changes were required.

Group discussions then focused on finalizing the variable definitions and establishing consensus on the causal links between them.

Phase 4: review and validation of the integrated SES map

Following the workshops, the research team conducted a thorough review and refinement of the socio-ecological maps. The steps included digitization of the maps using Cmaps software [5] under the supervision of an experienced modeler (SR). The refinement involved clarifying variable names for consistency (e.g., merging overlapping terms such as "animal health monitoring" and "veterinary surveillance"), ensuring variables were defined in quantifiable or observable terms, and removing redundant or non-logical links (e.g., duplicated or circular connections without causal meaning). No new variables were added, and all modifications were cross-checked against the original workshop material to maintain the authenticity of participants' inputs.

References

[1] Hovmand PS. Community Based System Dynamics. Springer; 2014.

[2] Scott RJ. Explaining how group model building supports enduring agreement. Journal of Management and Organization 2019;25:783–806. https://doi.org/10.1017/jmo.2017.12.

[3] Hovmand PS, Andersen DF, Rouwette E, Richardson GP, Rux K, Calhoun A. Group model-building "scripts" as a collaborative planning tool. Syst Res Behav Sci 2012;29:179–93. https://doi.org/10.1002/sres.2105.

[4] Uleman JF, Stronks K, Rutter H, Arah OA, Rod NH. Mapping complex public health problems with causal loop diagrams. Int J Epidemiol 2024;53. https://doi.org/10.1093/ije/dyae091.

[5] Cañas AJ, Hill G, Carff R, Suri N, Lott J, Gómez G, et al. CMAPTOOLS: A KNOWLEDGE MODELING AND SHARING ENVIRONMENT. 2004.

Workshop sites, governance level and dates

Site	Governance level	Dates
Kinshasa	National level	23-24 November 2022
Kinshasa	Provincial level	23-24 November 2022
Kinshasa	Communal level	03-04 April 2023
Equateur	Provincial level	28-29 August 2023
Kinshasa	National level/CCUS	10-11 August 2023
